# Libman-Sacks Endocarditis as the Initial Presentation of Systemic Lupus Erythematosus and Antiphospholipid Syndrome: A Multisystem Diagnostic Challenge

**DOI:** 10.7759/cureus.106058

**Published:** 2026-03-29

**Authors:** Swaid R Saulat, AbdulMohsen L AlSolami, Yasser S Gabr

**Affiliations:** 1 Emergency Department, Security Forces Hospitals Program, General Directorate of Medical Services, Ministry of Interior, Riyadh, SAU; 2 Emergency Department, Security Forces Hospital, Riyadh, SAU

**Keywords:** anticoagulation dilemma, antiphospholipid syndrome, autoimmune hemolytic anemia, diffuse alveolar hemorrhage, libman-sacks endocarditis, mitral regurgitation, multisystem involvement, plasma exchange, systemic lupus erythematosus, valvular heart disease

## Abstract

Libman-Sacks endocarditis (LSE), characterized by sterile valvular vegetations, is a recognized complication of systemic lupus erythematosus (SLE) and antiphospholipid syndrome (APS). However, its occurrence as the initial manifestation of previously undiagnosed autoimmune disease is uncommon and may create significant diagnostic uncertainty. A 27-year-old female presented to the emergency department with acute-onset exertional dyspnea, cough, and severe anemia, with a history of three consecutive first-trimester miscarriages. Initial evaluation revealed severe mitral regurgitation with valvular masses, raising suspicion for infective endocarditis. Further workup demonstrated elevated inflammatory markers, autoimmune hemolytic anemia, nephrotic-range proteinuria, and positive autoimmune serology, including antinuclear antibodies, anti-double-stranded DNA, anticardiolipin IgG, anti-β2-glycoprotein I IgG, and lupus anticoagulant. Transthoracic echocardiography showed severe mitral regurgitation with valvular masses and reduced left ventricular ejection fraction, while transesophageal echocardiography confirmed Libman-Sacks vegetations. Computed tomography of the chest revealed diffuse alveolar hemorrhage. The patient was diagnosed with new-onset SLE and APS, presenting with LSE and multisystem involvement. Management included pulse methylprednisolone therapy, hydroxychloroquine, cyclophosphamide, therapeutic anticoagulation, and plasma exchange for alveolar hemorrhage, resulting in clinical improvement with resolution of hemoptysis and improvement in cardiac function. This case is particularly notable because severe valvular disease led to the initial recognition of previously undiagnosed SLE and APS in the setting of simultaneous pulmonary, hematologic, renal, and obstetric manifestations. It highlights the importance of considering autoimmune etiologies in young patients with unexplained valvular heart disease, particularly in the presence of suggestive systemic features.

## Introduction

Libman-Sacks endocarditis (LSE), first described in 1924, is a noninfectious thrombotic valvulopathy characterized by sterile vegetations that most commonly involve the mitral and aortic valves [1]. It is classically associated with systemic lupus erythematosus (SLE) and antiphospholipid syndrome (APS), in which immune-mediated endothelial injury and thrombus formation contribute to valvular pathology [2,3]. Although LSE is usually identified in patients with established autoimmune disease, presentation as an initial manifestation of SLE or APS is uncommon and poses a significant diagnostic challenge [2].

APS is defined by characteristic clinical manifestations, including thrombosis and pregnancy morbidity, together with persistent antiphospholipid antibodies [4]. Antiphospholipid antibodies contribute to disease through endothelial activation, complement pathway engagement, and interference with endogenous anticoagulant mechanisms [5]. Cardiac involvement in APS may include valvular thickening, regurgitation, and nonbacterial vegetations, while severe multisystem inflammation in SLE may further complicate the presentation [5,6]. Diffuse alveolar hemorrhage is a serious and potentially life-threatening pulmonary manifestation of SLE and may occur in the setting of severe systemic disease [7].

LSE may remain clinically silent and be detected incidentally on echocardiography; when it is symptomatic, it can present with cardiac murmurs, valvular regurgitation, heart failure symptoms, or systemic embolic phenomena such as stroke [1,6,8]. In these scenarios, the clinical and echocardiographic features may closely resemble infective endocarditis, degenerative valvular disease, or intracardiac masses, necessitating a comprehensive and systematic diagnostic approach to establish the correct etiology [3].

This report describes a complex case of new-onset SLE and APS presenting with LSE complicated by severe mitral regurgitation, diffuse alveolar hemorrhage, autoimmune hemolytic anemia, and lupus nephritis. What makes this case particularly notable is that severe valvular disease led to the initial recognition of previously undiagnosed SLE and APS in the setting of simultaneous pulmonary, hematologic, renal, and obstetric manifestations. The case highlights the diagnostic considerations, therapeutic approach, and clinical outcome in a young patient with extensive multisystem involvement.

## Case presentation

A 27-year-old nulliparous female presented to the emergency department with progressive exertional dyspnea corresponding to New York Heart Association class II, palpitations, and a nonproductive cough of one week’s duration. She also reported three consecutive first-trimester pregnancy losses within the preceding year, with the most recent occurring four weeks before presentation. She denied fever, chest pain, arthralgia, skin rash, oral ulcers, or photosensitivity. Her medical history was notable only for childhood asthma with poor medication adherence, and there was no family history of autoimmune, thrombotic, or hematologic disorders.

On initial assessment, the patient appeared pale. Vital signs revealed a blood pressure of 160/106 mmHg, heart rate of 120 beats per minute, respiratory rate of 18 breaths per minute, temperature of 36.6 °C, and oxygen saturation of 98% on room air. Cardiovascular examination demonstrated tachycardia with a regular rhythm, a soft first heart sound, and a loud holosystolic murmur graded 4/6 at the mitral area with radiation to the axilla. Bibasilar crackles were present on pulmonary auscultation. There was no peripheral edema, synovitis, skin rash, lymphadenopathy, or oral ulceration.

Initial laboratory evaluation revealed severe anemia with hemolytic features, progressive thrombocytopenia, acute kidney injury, and elevated inflammatory markers. Autoimmune serologic testing demonstrated positive antinuclear antibodies, anti-double-stranded DNA antibodies, lupus anticoagulant, anticardiolipin IgG, and anti-β2-glycoprotein I IgG antibodies, supporting the diagnoses of SLE and APS [4]. Nephrotic-range proteinuria was also identified, raising concern for lupus nephritis.

**Figure 1:**
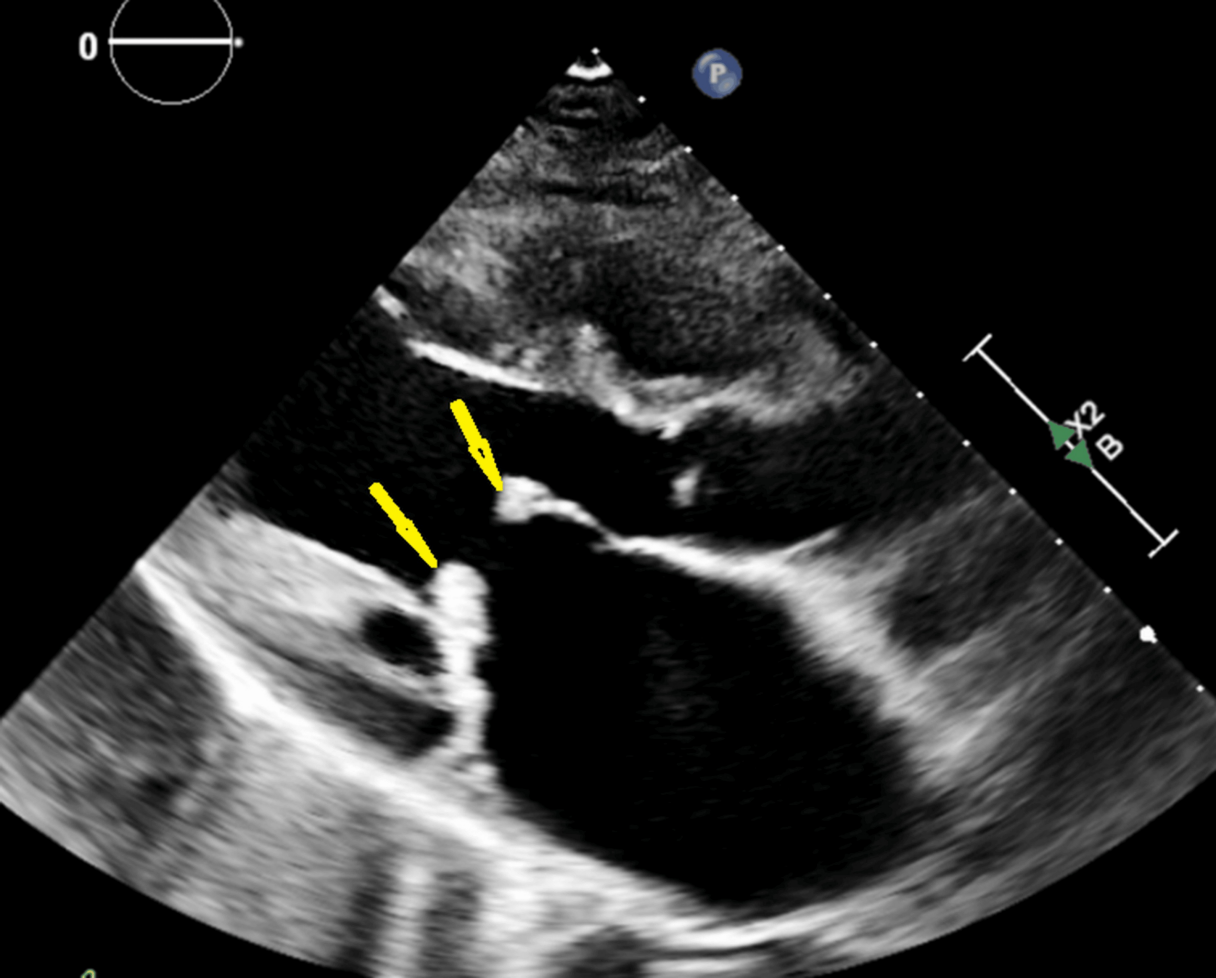
Transthoracic echocardiography at presentation Transthoracic echocardiography demonstrating thickened mitral valve leaflets with irregular echogenic masses on the atrial surfaces (yellow arrows), associated with severe mitral regurgitation, consistent with Libman-Sacks endocarditis at presentation.

Transthoracic echocardiography demonstrated thickened mitral valve leaflets with echogenic masses measuring approximately 8-10 mm on both leaflets, severe mitral regurgitation with a vena contracta of 6.5 mm, severe left atrial dilation measuring 51 mm, reduced left ventricular ejection fraction of 45%, and severe pulmonary hypertension with an estimated pulmonary artery systolic pressure of 60-65 mmHg. Transesophageal echocardiography confirmed these findings, demonstrating kissing masses on the atrial surfaces of both mitral leaflets, consistent with LSE (Figure 1).

Because the initial valvular findings raised concern for infective endocarditis, the differential diagnosis was evaluated systematically. Three sets of blood cultures were obtained on the day of presentation before completion of the diagnostic workup, and there was no documented antibiotic exposure before the initial culture collection. All initial blood culture sets remained negative at five days. The patient was afebrile, had no convincing clinical source of infection, and the subsequent autoimmune and antiphospholipid workup supported a noninfective autoimmune valvulopathy. In brief Duke-International Society for Cardiovascular Infectious Diseases (ISCVID) terms, although echocardiography demonstrated valvular masses, there was no major microbiologic criterion, and the overall clinical picture favored LSE rather than definite infective endocarditis [9].

**Figure 2:**
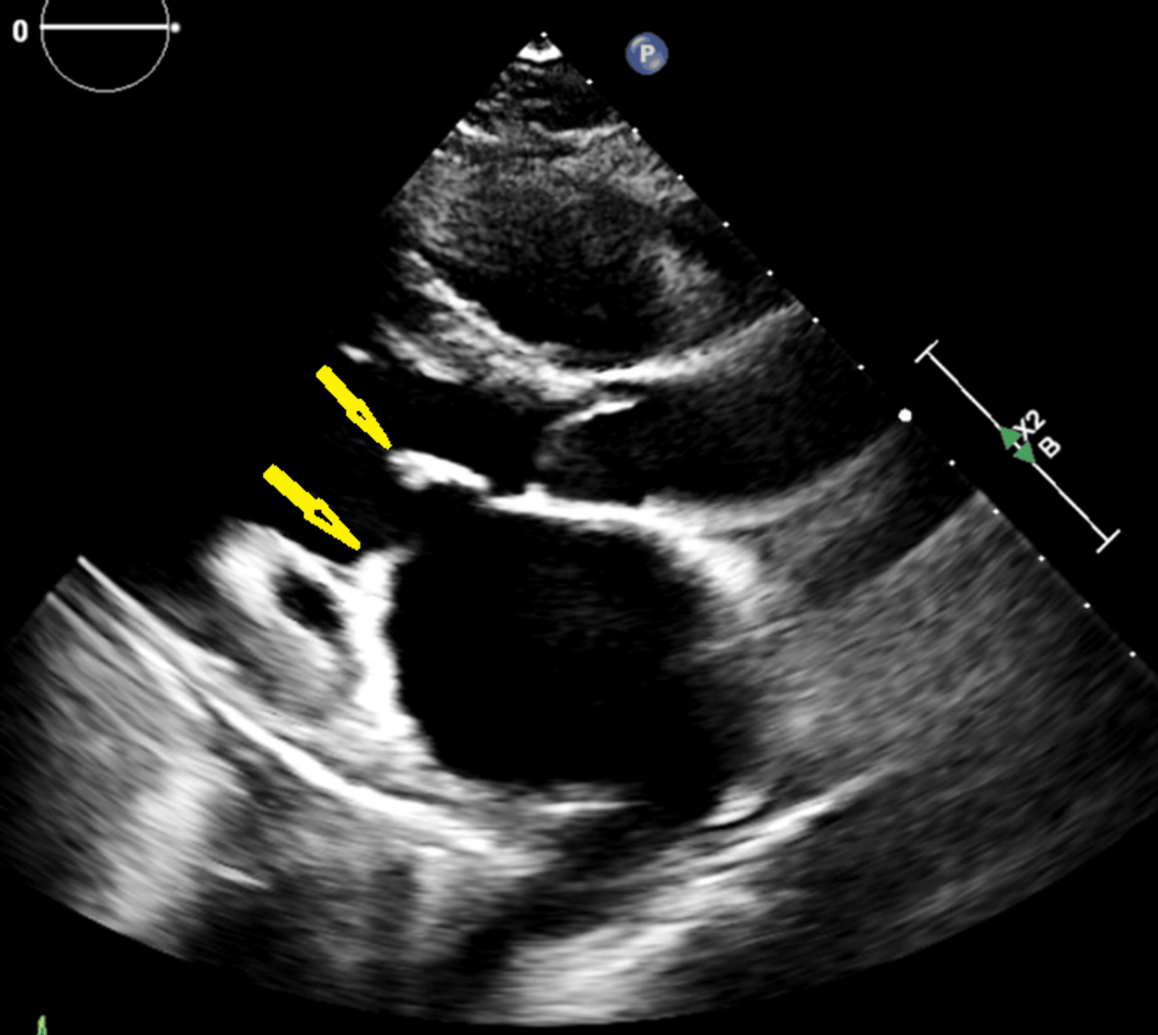
Follow-up transthoracic echocardiography Follow-up transthoracic echocardiography demonstrating persistent mitral valve vegetations (yellow arrows) with partial improvement in left ventricular systolic function following immunosuppressive therapy.

Follow-up transthoracic echocardiography performed after initiation of immunosuppressive therapy demonstrated persistent mitral valve vegetations with partial improvement in left ventricular systolic function (Figure 2).

**Figure 3:**
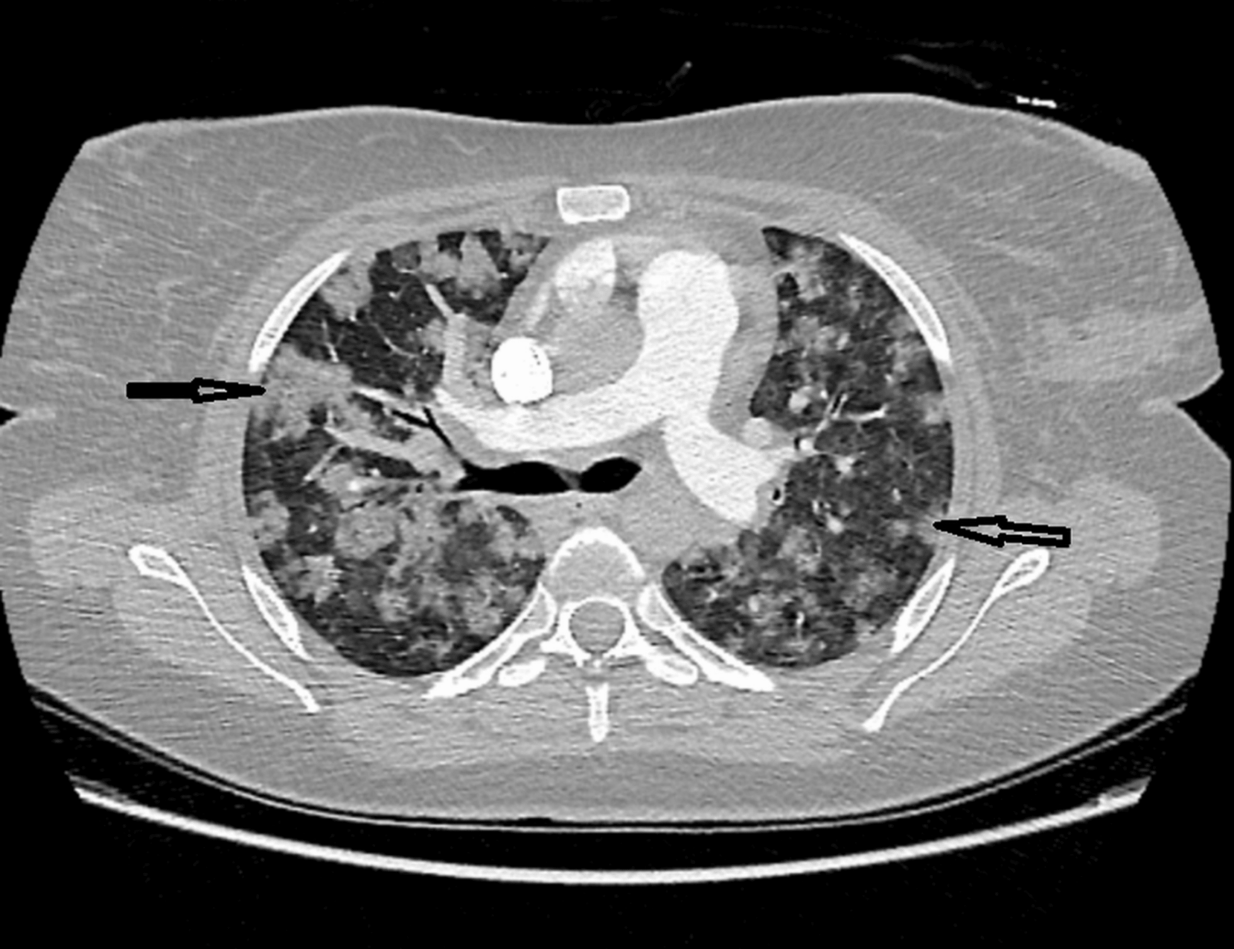
Computed tomography of the chest Axial contrast-enhanced computed tomography scan of the chest demonstrating bilateral diffuse ground-glass opacities with a crazy-paving pattern (arrows), consistent with diffuse alveolar hemorrhage.

Computed tomography pulmonary angiography excluded pulmonary embolism but revealed diffuse bilateral ground-glass opacities with a crazy-paving pattern. During hospitalization, the patient developed hemoptysis requiring admission to the intensive care unit. The imaging findings and clinical presentation were consistent with diffuse alveolar hemorrhage (Figure 3).

Management was coordinated through a multidisciplinary approach and included pulse methylprednisolone therapy followed by high-dose oral corticosteroids with gradual tapering, hydroxychloroquine, and cyclophosphamide according to the National Institutes of Health regimen. Therapeutic anticoagulation was initiated with heparin and later transitioned to warfarin with a target international normalized ratio of 2.5-3.5, with careful monitoring because of concurrent pulmonary hemorrhage. The patient also underwent five sessions of plasma exchange and received supportive care, including blood transfusion, diuretics, afterload reduction with angiotensin-converting enzyme inhibitors, and beta-blockade.

Clinical improvement was observed within 72 hours of initiating therapy, with resolution of hemoptysis, improvement in dyspnea, and normalization of inflammatory markers. Follow-up transthoracic echocardiography demonstrated partial improvement in left ventricular systolic function, with an ejection fraction of 52%, although severe mitral regurgitation persisted. The patient was discharged on hospital day 18 with plans for continued outpatient immunosuppressive therapy and serial echocardiographic monitoring.

## Discussion

This case highlights important clinical and pathophysiological aspects of SLE and APS presenting with LSE. The initial presentation closely resembled infective endocarditis, prompting extensive microbiological evaluation, including three sets of blood cultures. However, despite the recognized possibility of culture-negative infective endocarditis, the patient remained afebrile, the initial blood cultures were negative, there was no convincing infectious source, and the autoimmune and antiphospholipid serologic profile strongly supported a noninfective etiology. The patient’s history of recurrent unexplained first-trimester pregnancy losses was an important clinical clue, raising early suspicion for underlying APS even before confirmatory laboratory testing was completed [4].

LSE is a thrombotic-inflammatory valvulopathy characterized by endothelial injury, immune complex deposition, and platelet-fibrin thrombus formation. In APS, antiphospholipid antibodies promote a hypercoagulable state through several mechanisms, including endothelial activation, complement pathway engagement, and interference with endogenous anticoagulant systems such as annexin A5 and protein C [5]. The coexistence of diffuse alveolar hemorrhage (DAH), although seemingly paradoxical in a prothrombotic disorder, is a recognized manifestation of severe SLE and reflects acute pulmonary capillaritis driven by intense immune-mediated inflammation. This combination underscores the complex and simultaneous interaction of thrombotic and inflammatory pathways in advanced autoimmune disease.

Management in this case was particularly challenging because of the need to balance anticoagulation for APS against the risk of worsening pulmonary hemorrhage. Therapeutic anticoagulation was temporarily withheld during active alveolar bleeding and was cautiously reintroduced after clinical stabilization with immunosuppressive therapy. The extent of multisystem involvement, including cardiac, pulmonary, renal, and hematologic manifestations, required aggressive immunosuppression with pulse corticosteroids, cyclophosphamide, and adjunctive plasma exchange to control disease activity and limit further organ damage. Surgical intervention for severe mitral regurgitation was deferred during the acute phase because of systemic inflammation and clinical instability, with the understanding that long-term follow-up would determine the eventual need for valve repair or replacement [6].

LSE remains an important source of morbidity in patients with SLE and APS, and a proportion of patients progress to clinically significant valvular dysfunction requiring surgical intervention [6]. DAH is associated with particularly poor outcomes and remains one of the most serious pulmonary complications of SLE [7]. The favorable response observed in this patient underscores the importance of early recognition, comprehensive diagnostic evaluation, and coordinated multidisciplinary management when severe valvular, pulmonary, hematologic, and renal manifestations occur together in the setting of previously undiagnosed autoimmune disease.

Limitations

This report has several limitations. It describes a single case and therefore does not allow for causal inference or generalization of findings. Some clinical details were extracted retrospectively from the medical record, and the relative contribution of individual therapeutic interventions cannot be determined. Although initial testing supported APS, confirmatory repeat testing at 12 weeks, as required for classification, was not performed. Bronchoscopy was not performed because of clinical instability; therefore, the diagnosis of diffuse alveolar hemorrhage was based on hemoptysis and imaging findings rather than bronchoscopic confirmation.

## Conclusions

This case illustrates that Libman-Sacks endocarditis may be an initial manifestation of systemic lupus erythematosus and antiphospholipid syndrome. In young patients presenting with unexplained valvular abnormalities, particularly when accompanied by obstetric history or evidence of multisystem involvement, autoimmune etiologies should be considered in the differential diagnosis. A structured diagnostic approach incorporating clinical history, autoimmune serology, and echocardiographic evaluation is important for timely diagnosis. This case also highlights the value of coordinated multidisciplinary management in complex presentations involving simultaneous cardiac, pulmonary, hematologic, and renal manifestations.
